# [^68^Ga]FAPI PET/CT reveals increased pulmonary fibroblast activation protein expression in long COVID patients after ICU discharge

**DOI:** 10.1007/s00259-025-07376-y

**Published:** 2025-06-21

**Authors:** B. van Leer, M. Londema, Ö. Kasalak, J. H. van Snick, M. L. Duiverman, J. C. Kuijvenhoven, M. D. de Kruif, D. E. Oprea-Lager, K. Pabst, M. E. Hellemons, H. H. Boersma, M. Prokop, M. W. Nijsten, A. W. J. M. Glaudemans, J. Pillay, R. H. J. A. Slart

**Affiliations:** 1 Department of Critical Care, University Medical Center Groningen, University of Groningen, Groningen, The Netherlands; 2 Department of Nuclear Medicine and Molecular Imaging, University Medical Center Groningen, University of Groningen, Hanzeplein 1, 9700 RB, TA29, PO box: 30001, Groningen, The Netherlands; 3 Department of Radiology, University Medical Center Groningen, University of Groningen, Groningen, The Netherlands; 4 Department of Pulmonology, University Medical Center Groningen, University of Groningen, Groningen, The Netherlands; 5 Department of Pulmonology, Medical Center Leeuwarden, Leeuwarden, The Netherlands; 6 Department of Pulmonology, Zuyderland Medical Center, Heerlen, The Netherlands; 7 Department of Radiology and Nuclear Medicine, Amsterdam University Medical Centers, Amsterdam, The Netherlands; 8 Department of Radiology and Nuclear Medicine, Radboud University Medical Center, Radboud University, Nijmegen, The Netherlands; 9 Department of Nuclear Medicine and Molecular Imaging, University Medical Center Essen, Essen, Germany; 10 Department of Pulmonology, Erasmus Medical Center, Erasmus University Rotterdam, Rotterdam, The Netherlands; 11 Department of Clinical Pharmacy and Pharmacology, University Medical Center Groningen, University of Groningen, Groningen, The Netherlands; 12 Department of Radiology, Radboud University Medical Center, Radboud University, Nijmegen, The Netherlands; 13 Groningen Research Institute for Asthma and COPD (GRIAC), University Medical Center Groningen, University of Groningen, Groningen, Netherlands; 14 Biomedical Photonic Imaging Group, Faculty of Science and Technology, University of Twente, Enschede, The Netherlands

**Keywords:** Post-acute sequalae of COVID-19, Long COVID, FAPI PET/CT, Critically ill, Respiratory, Muscles

## Abstract

**Purpose:**

Post-acute sequelae of COVID-19 (PASC) has emerged as a major healthcare problem. A comprehensive mechanism of disease remains to be elucidated. In this study we aimed to explore pulmonary and muscle fibroblast activation protein (FAP) activity in former critical COVID-19 patients with persistent dyspnea, using [^68^Ga]FAPI-46 PET/CT.

**Methods:**

In this single center prospective observational study we included former critical COVID-19 patients reporting complaints of dyspnea > 3 months after hospital discharge. A [^68^Ga]FAPI PET/CT scan was performed including a high-resolution CT scan, lung function test, EQ-5D questionnaire, 6 min walking test and inflammatory markers. Age and sex-matched subjects, without pulmonary pathology, served as controls. The [^68^Ga]FAPI uptake was corrected for lean body mass and the target-to-background ratio (TBR) was calculated.

**Results:**

Eighteen PASC patients and 15 controls (median age 59 and 63 years and BMI of 34.6 and 25.2 kg/m^2^) were included. The interval between hospital discharge and study visit was 30 months. Increased pulmonary FAP expression was observed in PASC, (TBR 0.79 ± 0.23) compared to controls (TBR 0.40 ± 0.13, *P* < 0.001). Increased FAP expression was also observed in the paravertebral muscles (PASC: TBR 1.17 and controls TBR 1.00, *P* = 0.03). Forced expiratory volume and forced vital capacity showed moderate negative correlation with the pulmonary TBR, while the percentage of ground glass opacities showed a moderate positive correlation.

**Conclusion:**

[^68^Ga]FAPI PET/CT demonstrated elevated FAP expression in PASC. These findings provide insight into possible pathophysiological mechanisms of PASC and a potential new diagnostic modality.

## Introduction

The syndrome of post-acute sequelae of COVID-19 (PASC) has emerged as a major healthcare problem [1]. Although several pathophysiological pathways have been proposed, a comprehensive mechanism of disease remains to be elucidated [2]. Therefore, to date, the diagnosis is based on unspecific self-reported symptoms only, hampering objective diagnosis and targeted therapy [3].

Complaints of dyspnea and fatigue are important phenotypes of PASC [4]. Imaging may aid in diagnosis and increase understanding of pulmonary abnormalities in these patients. Although studies show variations in the occurrence of abnormalities on chest computed tomography (CT), this modality has not provided definitive diagnostic or pathophysiological hallmarks [5, 6].

The proposed pathophysiology of PASC includes persistent low-grade inflammation and microthrombosis [7]. Both mechanisms can contribute to fibroblast activation. Molecular imaging with ^68^Ga-fibroblast activation protein inhibitor ([^68^Ga]FAPI) positron emission tomography (PET) allows for whole body assessment of in vivo fibroblast activation. [^68^Ga]FAPI binds to fibroblast activation protein (FAP) which is expressed on activated fibroblasts [8]. Our aim was to explore pulmonary FAP activity in former critical COVID-19 patients, with persistent complaints of dyspnea. We hypothesised that PASC patients show a higher pulmonary [^68^Ga]FAPI uptake compared to controls.

## Methods

A prospective observational study was conducted in PASC patients, reporting complaints of dyspnea > 3 months after hospital discharge for confirmed SARS-CoV-2 infection, requiring mechanical ventilation or high-flow nasal oxygen therapy. Patients with Chronic Obstructive Pulmonary Disease (COPD) were excluded. The study protocol was approved by the Institution Review Board of the University Medical Center Groningen (202100802) and registered at ClinicalTrials.gov (NCT05981885). All patients provided written informed consent.

### Imaging

Each subject underwent a PET and low-dose CT (PET/CT) scan (Biograph Vision 600 or Biograph Vision Quadra, Siemens Healthineers, Knoxville, TN, USA), from head to proximal femur 60 min following intravenous injection of 200 MBq (range 150–250 MBq) [^68^Ga]FAPI-46 (SOFIE, Dulles, Virginia, USA). Scan time on the Quadra was 3 min in one bed position and on the Vision 20 min in continuous bed motion. At the time of the study, respiratory gating was not available. Image reconstruction was performed according to updated EANM Research Ltd. (EARL) specifications, EARL2 [9]. High-Resolution CT (HRCT) scan of the lungs in inspiration was performed on the same scanner.

### Image segmentation

For quantification of the [^68^Ga]FAPI uptake the low-dose CT was segmented using automated segmentation software (TotalSegmentator 2.0.4, University Hospital Basel, Basel, Switzerland) to create volumes of every lung lobe and paravertebral muscles [10]. The obtained segmentations were mapped over the PET data and edited manually in HERMIA Affinity viewer 4.0 (Hermes Medical Solutions, Stockholm, Sweden) to extract spillover uptake from possible intercostal muscle uptake, liver and bone degeneration. Images were visually examined and uptake was classified as spillover when it followed the anatomical pattern of adjacent tissue. Only minor manual corrections were needed for incorrectly performed segmentations, mainly due to mismatch between right middle and lower lobe segmentations. To measure the background signal a 1 cm^3^ volume of interest (VOI) was placed in the blood pool of the vena cava inferior (VCI).

### [^68^Ga]FAPI quantification

**Fig. 1:**
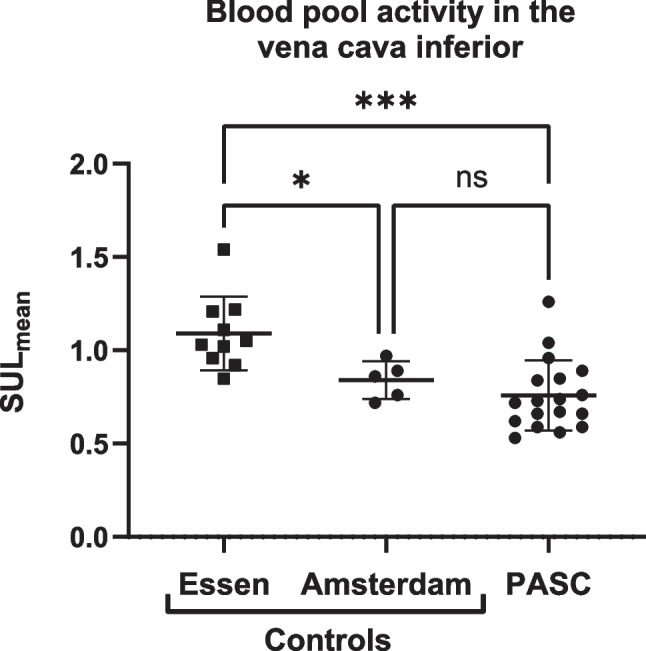
Blood pool activity of [^68^Ga]FAPI-46 per site. Bars represent median and inter quartile range (IQR). The significant higher blood pool activity in the patients of Essen is most likely explained by the difference in time between tracer administration and PET/CT. The scan time after injection of [^68^Ga]FAPI was between 10 and 25 min in Essen, ± 120 min in Amsterdam and ± 60 min in PASC patients. *SUL *standardized uptake value corrected for lean body mass, *PASC *post-acute sequelae of COVID-19. * = *P* of 0.02; *** = *P* < 0.001; ns = non-significant

The [^68^Ga]FAPI uptake was measured as standardized uptake value (SUV) corrected for lean body mass (SUL) (instead of only body weight) following the Janmahasatian method [11]. Next, to compare the [^68^Ga]FAPI uptake between patients, average SUL_mean_ uptake over all 5 lobes was computed, resulting in whole lung uptake. To measure the diffuse (homogeneous, non-focal) lung uptake, focal [^68^Ga]FAPI uptake areas were extracted by segmenting the < 50% isocontour of the SUV_max_ of the lung lobes. To compare the paravertebral muscle uptake the average of both SUL_mean_ values where calculated. The maximum uptake within both paravertebral muscles was obtained by selecting the highest SUL_max_ of both muscles. Target-to-background ratios (TBR) were calculated utilizing the VCI SUL_mean_ to account for interpatient and interinstitutional variability caused by differences in imaging protocol and tracer clearance (Fig. 1).

### Controls

Within the scope of this exploratory study it was considered to be unethical to expose healthy controls to additional radiation. Therefore, control subjects without pulmonary pathology, recruited from two different centres (Amsterdam University Medical Center, the Netherlands and University Medical Center Essen, Germany), scanned with [^68^Ga]FAPI-46 for oncological reasons, using similar camera systems, settings and analyses were included. In Amsterdam patients were scanned ± 2 h after tracer administration, in Essen ± 15 min (Fig. 1).

### HRCT

HRCTs were analyzed visually. Each lung lobe was scored for the presence of ground glass opacities and traction bronchiectasis. The presence of ground glass opacities was quantified utilizing a percentage score per lung lobe (0–100% of the lobe affected by ground glass opacities) and a 5-point CT severity scale for the whole lung (CTSS, each lobe is scored 0–5 based on the extent of lung involvement, with a maximal possible score of 25) [12].

### Patient characteristics

BMI, lung function and diffusion, 6 min walking test (6MWT), EQ-5D questionnaire, CRP, ESR and leukocytes were used for patient characterization.

### Statistical analysis

The independent samples t-test was used for comparison of the mean whole lung and diffuse [^68^Ga]FAPI uptake corrected for the blood pool (TBR_wl_ and TBR_diff_) between PASC patients and controls. To assess differences between institutions, an Brown-Forsythe and Welch ANOVA test was conducted (required applicable conditions were met). The correlation between clinical parameters and TBR_wl_ was explored using Pearsons or Spearman test. A post-hoc analysis comparing the [^68^Ga]FAPI paravertebral muscle uptake corrected for the blood pool between PASC patients and controls was performed after visual assessment suggested increased uptake in PASC patients. All analyses were two-sided except for the post-hoc muscle analysis, which was conducted using a one-sided approach, as our hypothesis of an increase in [^68^Ga]FAPI uptake was confirmed based on the lung measurement. A p-value < 0.05 was considered significant.

## Results

Eighteen PASC patients with a median age of 59 years were included. Patient characteristics are shown in Table 1. Ten were male (56%). None of the participants were active smokers. One patient had a medical history of asthma, another had obstructive sleep apnea requiring nightly continuous positive airway pressure (CPAP) ventilation, while none of the other patients had a history of pulmonary conditions. Median (IQR) BMI was 34.6 kg/m^2^ (31.1 to 42.4). The median interval between hospital discharge and study visit was 30 months (7.5 to 34.25). Sixteen patients (89%) were mechanically ventilated during their ICU admission, two received high-flow nasal oxygen therapy only. The median (IQR) involvement of ground glass opacities per lung lobe was 5% (0–10). The highest involvement of ground glass opacities for a single lobe was 25%. Nine patients (50%) showed 5% or less ground glass opacities in all lobes. Overall, lung function parameters were within normal limits in most patients (median percentage of predicted diffuse capacity for carbon monoxide (DCLO) was 79% (66 to 89), forced expiratory volume (FEV1) 90% (81 to 104) and forced volume capacity (FVC) 86% (74 to 92)). Median walking distance during the 6MWT was 393 meters (228 to 482). Dyspnea and fatigue reflected by the Borg scores worsened respectively 2 and 1 point after the 6MWT. The median EQ-5D index and EQ-VAS, representing subjective health status, were 0.77 (0.38 to 0.84) and 67.5 (50.0 to 80.0), respectively. Clinical characteristics are presented in Table 2.

**Table 1:** Patient and scan characteristics

	PASC *n* = *18*	Controls *n* = *15*	Sig
Age, *years*	59 [54 – 67]	63 [59 – 68]	.32
Male	10 (56)	9 (60)	.80
BMI, *kg/m*^*2*^	34.6 [31.1 – 42.4]	25.2 [22.4 – 26.8]	<.001
Smoking status			
Never	8 (44)		
Stopped	10 (56)		
Packyears	13.0 [4.3 – 19.5]		
Time between discharge and PET/CT, *months*	30 [8 – 33]		
Oxygen therapy during ICU admission			
Mechanical ventilation	16 (89)		
High-Flow Nasal Oxygen	2 (11)		
PET/CT characteristics			
Administered dose, *MBq*	190 [170 – 200]	143 [97 – 185]	0.1
Scanner type			
Siemens Vision Quadra	15 (83)	5 (33)	
Siemens Vision	3 (17)	10 (66)	

Median [Inter Quartile Range] or *n* (%). T-test or ANOVA used for continuous data, chi-square for bivariate data

**Table 2:** Clinical characteristics

Inflammatory markers	
CRP	1.8 [1.0 – 5.0] *mg/L*
ESR	18.5 [6.0 – 34.5] *mm/hr*
Leukocytes	7.8 [5.3 – 8.4] *10*^*9*^*/L*
Lung function	
DCLO	6.6 [5.2 – 7.6] mmol/(min*kPa)
DCLO %pred	79 [66 – 89] *%*
DCLO Z-score	−1.39 [−2.68 – −0.68]
FEV1	3.0 [2.5 – 3.4] *L*
FEV1%pred	90 [81 – 104] *%*
FEV1 Z-score	−0.72 [−1.2 – 0.23]
FVC	3.6 [2.9 – 4.3] *L*
FVC %pred	86 [74 – 92] *%*
FVC Z-score	−0.95 [−1.84 – −0.54]
FEV1/FVC	83.5 [81.0 – 87.0]
FEV1/FVC %pred	107 [103 – 112] *%*
FEV1/FVC Z-score	0.97 [0.43 – 1.37]
EQ-5D-5L questionnaire	
EQ-5D index	0.765 [0.383 – 0.841]
EQ-5D VATS	67.5 [50.0 – 80.0]
6MWT	
Distance	393.0 [227.5 – 482.0] *meters*
Difference Borg scale fatigue	1 [0 – 2]
Difference Borg scale dyspnea	2 [1 – 2]
Difference SpO2	0.5 [0.0 – 3.0] *%*
HRCT	
CT severity score (whole lung)	10 [2 – 10]
% of GGO (per lung lobe)	5 [0 – 10] *%*
Patients with all lobes ≤ 5% GGO	9 (50)
Lobes with traction bronchiectasis	1 [0 – 3]
Patients with no traction bronchiectasis	7 (39)

Median [Inter Quartile Range] or *n* (%). *BMI* body mass index, *CRP* C-reactive protein, *ESR* erythrocyte sedimentation rate, *DCLO* diffuse capacity for carbon monoxide, *FEV1* forced expiratory volume in 1 second, *FVC* forced vital capacity, %*pred* percentage of predicted, *6MWT* 6 min walking test, *HRCT* high-resolution computed tomography, *GGO* ground glass opacities

In total, 15 age- and gender-matched control patients were included. However, BMI was higher in the PASC group compared to controls (*P* < 0.001) (Table 1).

**Fig. 2:**
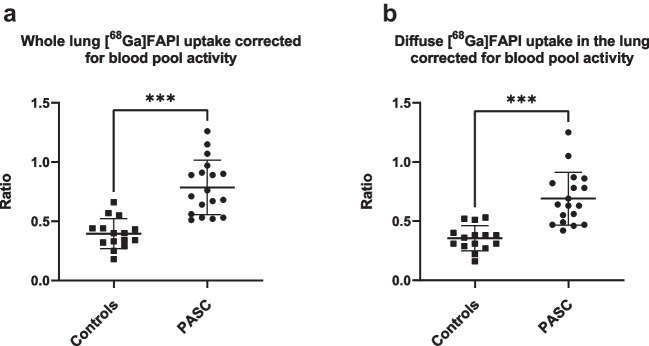
[^68^Ga]FAPI uptake in the lungs of PASC patients vs controls. Bars represent mean and standard deviation. **A** Whole lung uptake (SUL_mean_) corrected for blood pool background uptake (TBR). **B** Diffuse (non-focal, homogeneous) whole lung uptake (SUL_mean_) corrected for blood pool background uptake (TBR). *SUL *standardized uptake value corrected for lean body mass, *TBR *target to background ratio, *PASC *post-acute sequelae of COVID-19. *** = *P* < 0.001

**Fig. 3:**
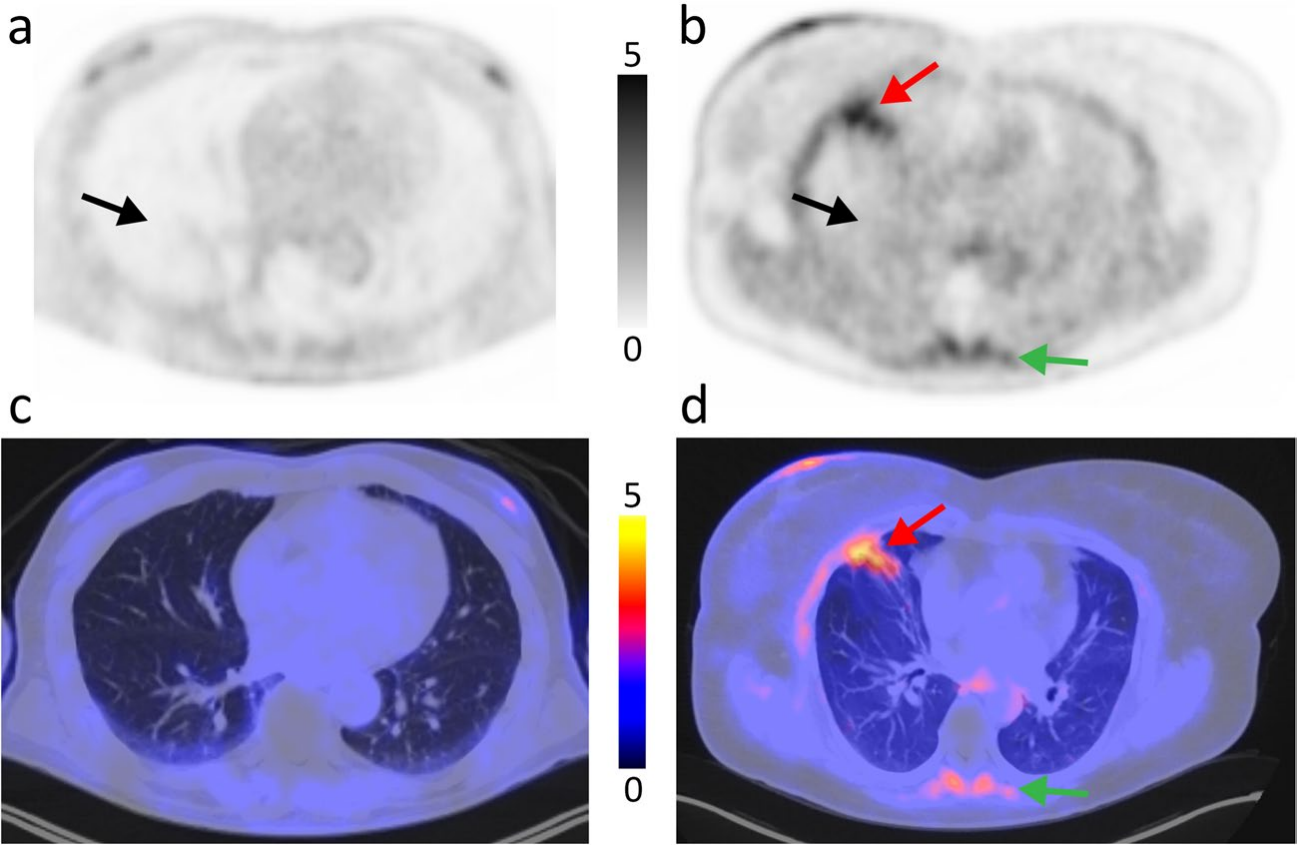
[^68^Ga]FAPI PET/CT scan of a PACS patient and control. EARL 2 reconstructed. **A** Transversal pulmonary PET image of a control patient. **B** Transversal pulmonary PET image of a PASC patient. **C** Transversal pulmonary fusion PET/CT image of a control patient. **D**. Transversal pulmonary fusion PET/CT image of a PASC patient. Black arrow: lung tissue, representative for diffuse [^68^Ga]FAPI uptake in the lungs. Red arrow: focal [^68^Ga]FAPI uptake in a fibrotic lesion. Green arrow: [^68^Ga]FAPI uptake in the paravertebral muscles. Settings: SUV 0–5, low-dose CT W 1200, C −600. Fusion 50/50

Increased pulmonary [^68^Ga]FAPI uptake (TBR_wl_) was observed in the PASC patients (mean TBR: 0.79 ± 0.23) compared to controls (mean TBR 0.40 ± 0.13, P < 0.001)(Fig. 2A). On visual inspection more diffuse pulmonary uptake was observed in PASC, we therefore compared only the diffuse uptake (excluding the patchy more focal uptake) (TBR_diff_) (Fig. 2B), resulting in a mean TBR ratio of 0.69 ± 0.22 for PASC patients and 0.36 ± 0.11 for controls (*P* < 0.001). Figure 3 shows an example of focal and diffuse uptake in a PASC patient compared to a control patient. Institutional comparison between Essen en Amsterdam showed no significant differences (TBR_wl_*P* = 0.46 and TBR_diff_*P* = 0.43).

Time between hospital discharge and [^68^Ga]FAPI PET/CT, FEV1 and FVC showed a moderate negative correlation with TBR_wl_ (*r* = −0.58, *P* = 0.01; r = −0.51, *P* = 0.03 and r = −0.54, *P* = 0.02, respectively). The percentage of ground glass opacities showed a moderate positive correlation with TBR_wl_ (r = 0.22 and *P* = 0.04). Other clinical parameters showed no correlation (Table 3).

**Table 3:** Correlation between pulmonary [^68^Ga]FAPI uptake (corrected for blood pool uptake) and clinical outcome

	Pearson/Spearman’s correlation coefficient	Sig
Time between [^68^Ga]FAPI PET/CT and hospital discharge	−0.58	0.01*
Inflammatory parameters		
CRP	0.12	0.64
ESR	0.29	0.36
Leukocytes	−0.11	0.67
Lung function		
DCLO %pred	−0.43	0.10
FEV1 %pred	−0.51	0.03*
FVC %pred	−0.54	0.02*
FEV1/FVC %pred	0.35	0.16
EQ-5D-5L questionnaire		
EQ-5D index	0.05	0.86
EQ-5D VATS	−0.07	0.79
6-MWT		
Distance	−0.24	0.33
Difference Borg scale fatigue	−0.10	0.70
Difference Borg scale dyspnea	0.42	0.09
HRCT		
CT severity score (whole lung)	0.33	0.18
% of GGO (per lung lobe)	0.37	< 0.001*

^*^ = significant *P*-value. *DCLO* diffusion capacity for carbon monoxide, *FEV1* forced expiratory volume in 1 second, *FVC* forced vital capacity, *%pred* percentage of predicted, *6-MWT* 6-min walking test; *ESR* erythrocyte sedimentation rate, *HRCT* high resolution computed tomography, *GGO* ground glass opacities

**Fig. 4:**
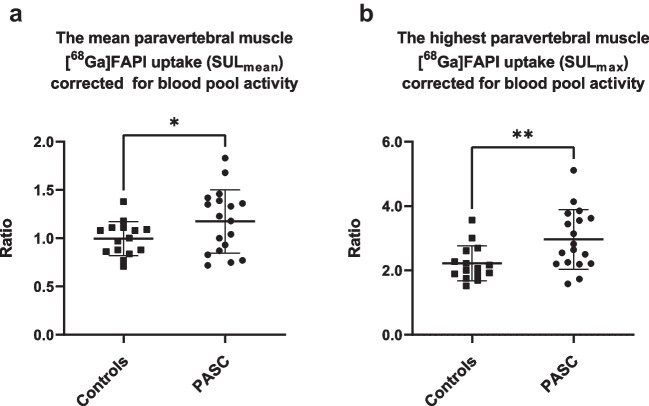
[^68^Ga]FAPI uptake in the paravertebral muscles of PASC patients and controls. Bars represent mean and standard deviation (SD). **A** Paravertebral muscle uptake (SUL_mean_) corrected for blood pool background uptake (TBR) (PASC: 1.2, SD ± 0.3 and controls: 1.0, SD ± 0.2). **B** Paravertebral muscle uptake (SUL_max_) corrected for blood pool background uptake (TBR) (PASC:2.9, SD ± 0.9 and controls: 2.2, SD ± 0.54). *SUL *standardized uptake value corrected for lean body mass, *PASC *post-acute sequelae of COVID-19. * = *P* of 0.03, ** = *P* of 0.004

Paravertebral muscle uptake was visually observed and post-hoc analyses showed significant difference between PASC and controls for the mean and maximal [^68^Ga]FAPI uptake corrected for blood pool activity (TBR PASC: 1.17 (± 0.33) and controls: 1.00 (± 0.17), *P* = 0.03 and TBR PASC: 2.9 (± 0.92) and controls: 2.2 (± 0.54), *P* = 0.004) (Fig. 4). The increase in fatigue following the 6MWT correlates with both the mean and maximal [^68^Ga]FAPI muscle uptake (r = 0.70, *P* = 0.002 and *r* = 0.65, *P* = 0.005). Whereas the increase of dyspnea after the 6 MWT, the EQ-index and the EQ-VATS did not correlate.

## Discussion

We explored the expression of FAP using [^68^Ga]FAPI-46 PET/CT in PASC patients after critical COVID-19. More than 2 years after hospital discharge, markedly increased pulmonary [^68^Ga]FAPI uptake was observed in PASC compared to controls. There was a moderate correlation of FEV1, FVC and percentage ground glass opacities with pulmonary [^68^Ga]FAPI uptake corrected for blood pool activity, however no other correlation between TBR_wl_ and clinical parameters was found. In addition, we found increased FAP expression in muscle tissue of PASC patients.

Pulmonary FAP expression, representing fibroblast activation, was first described in a former COVID-19 patient showing pulmonary [^68^Ga]FAPI uptake matching ground glass opacities on HRCT [13]. These findings were confirmed in a small prospective study including 6 PASC patients and 9 controls [14]. However, this study only included patients with persistent CT abnormalities, which are frequently absent in PASC [5]. In this study, we found increased long-term pulmonary FAP expression, only moderately correlated to the presence of ground glass opacities. Supporting the finding that not all PASC patients presents pronounced CT abnormalities [5].

The lung function of the PASC patients showed a mild restrictive pattern, with a lower FVC and a slightly higher FVC/FEV1 than predicted. Although FVC and FEV1 correlated mildly with [^68^Ga]FAPI uptake, FVC/FEV1 did not. Pulmonary FAP expression has been shown to be elevated in restrictive lung diseases [15–17]. However, this small exploratory study did not allow for correction for confounders, which are linked to FVC and FEV1, and therefore could not establish causality between FAP expression and any clinical parameters.

To our knowledge this is the first time that increased muscle FAP expression in PASC patients has been shown. Skeletal muscle involvement in the presence of exertion related PASC symptoms has been described [18]. Future research should verify these findings and focus on the contribution of muscle FAP expression in specific PASC symptoms. Especially since non-pathological (focal) muscle uptake has been described [19, 20].

Elevated FAP expression suggests ongoing tissue remodelling in PASC with persistent respiratory complaints, therefore, antifibrotic drugs might be promising [7, 21, 22]. However, it is currently unknown which PASC patients might benefit most from these therapies. The absence of a comprehensive mechanism of disease leads to high heterogeneity of the study population, which may contribute to negative study outcomes. Differentiating between FAP-positive and FAP-negative PASC patients with [^68^Ga]FAPI PET/CT may enhance subject selection and reduce heterogeneity, and make therapy more effective. [^68^Ga]FAPI PET/CT may aid in monitoring therapy response and guiding treatment decisions.

While our study provides valuable insights, several limitations must be acknowledged. First, it concerns a small exploratory study. However, this is the only study to date to include FAPI imaging corrected for BMI, combined with clinical parameters. Secondly, including PASC patients with a history of critical COVID-19 and persistent respiratory complaints precluded generalization to non-hospitalized PASC patients. Thirdly, the lack of motion correction necessitated to perform manual correction from spill-over from the adjacent muscles and liver, introducing observer variability and reducing reproducibility. However, the measured diffuse pulmonary uptake is less likely to be influenced by the manual corrections as we subtracted these higher uptake areas. The difference in FAP expression between PASC patients and controls remained. Finally, this study included controls scanned at other institutions and limited medical information on co-morbidities and treatment of these controls was available. Furthermore, patients were scanned at different time intervals between image acquisition and tracer administration. However, blood pool activity of FAPI stabilizes quickly after the first 10 to 20 min. [23, 24] After correcting for blood pool activity there was no significant difference in pulmonary [^68^Ga]FAPI uptake between the patients from Essen (shortest time interval) and those from Amsterdam (longest time interval). Minimizing potential differences caused by isotope preparation or scanning protocols. Furthermore, pulmonary [^68^Ga]FAPI uptake in our controls were consistent with the (healthy) controls of previous studies [16, 17].

A follow-up study will be conducted (NCT06911476) in former non-severe COVID-19 patients with PASC to generalize our results. This future study will include recovered PASC patients as controls. All participants will be scanned on the same scanner within the same institution to fully eliminate potential institutional differences.

The batch of FAPI-46 precursor used in this study was identified by GE HealthCare (license holder of FAPI-46) to contain approximately 20% of the non-binding (R)-enantiomer, resulting in a deviation from the expected > 98% enantiomeric purity of the active (S)-enantiomer. While the (R)-enantiomer is pharmacologically inactive and does not bind to the target, its presence may have contributed to a modest increase in non-specific uptake. If the increase of non-specific uptake has influenced our results it has most likely resulted in a lower target-to-background ratio. A detailed analysis of the different enantiomeric compositions and its implications will be described in a forthcoming publication by GE HealthCare. Importantly, the administered amount of the (R)-enantiomer was very low (< 10 µg), and no safety concerns were observed during the study.

## Conclusion

[^68^Ga]FAPI PET/CT scan demonstrated elevated FAP expression in PASC. These findings provide insight into possible pathophysiological mechanisms of PASC and a potential new diagnostic modality.

## Data Availability

Fully anonymized data supporting the conclusion of this article will be made available by the authors on reasonable request.

## References

[1] Ballering AV, van Zon SKR, olde Hartman TC, Rosmalen JGM. Persistence of somatic symptoms after COVID-19 in the Netherlands: an observational cohort study. Lancet. 2022 [cited 2024 Jun 26];400:452–61. 10.1016/S0140-6736(22)01214-4. 10.1016/S0140-6736(22)01214-4 PMC9352274 35934007

[2] Hodgson CL, Broadley T. Long COVID—unravelling a complex condition. Lancet Respir Med. 2023 [cited 2024 Feb 23];11:667–8. 10.1016/S2213-2600(23)00232-1. 10.1016/S2213-2600(23)00232-1 37475126

[3] Thaweethai T, Jolley SE, Karlson EW, Levitan EB, Levy B, McComsey GA, et al. Development of a definition of postacute sequelae of SARS-CoV-2 infection. JAMA. 2023 [cited 2024 Apr 23];329:1934–46. 10.1001/JAMA.2023.8823. 10.1001/jama.2023.8823 PMC10214179 37278994

[4] Gentilotti E, Górska A, Tami A, Gusinow R, Mirandola M, Rodríguez Baño J, et al. Clinical phenotypes and quality of life to define post-COVID-19 syndrome: a cluster analysis of the multinational, prospective ORCHESTRA cohort. EClinicalMedicine. 2023 [cited 2024 Feb 23];62. 10.1016/j.eclinm.2023.102107. 10.1016/j.eclinm.2023.102107 PMC10466236 37654668

[5] Bazdar S, Kwee AKAL, Houweling L, de Wit-van Wijck Y, Mohamed Hoesein FAA, Downward GS, et al. A Systematic Review of Chest Imaging Findings in Long COVID Patients. J Pers Med. 2023 [cited 2024 Feb 23];13. 10.3390/JPM13020282/S1. 10.3390/jpm13020282 PMC9965323 36836515

[6] Babar M, Jamil H, Mehta N, Moutwakil A, Duong TQ. Short- and long-term chest-CT findings after recovery from COVID-19: a systematic review and meta-analysis. Diagnostics. 2024 [cited 2024 Apr 23];14:621. 10.3390/DIAGNOSTICS14060621/S1. 10.3390/diagnostics14060621 PMC10969005 38535041

[7] Singh SJ, Baldwin MM, Daynes E, Evans RA, Greening NJ, Jenkins RG, et al. Respiratory sequelae of COVID-19: pulmonary and extrapulmonary origins, and approaches to clinical care and rehabilitation. Lancet Respir Med. 2023 [cited 2024 Feb 21];11:709–25. 10.1016/S2213-2600(23)00159-5. 10.1016/S2213-2600(23)00159-5 PMC10198676 37216955

[8] Loktev A, Lindner T, Mier W, Debus J, Altmann A, Jäger D, et al. A Tumor-Imaging Method Targeting Cancer-Associated Fibroblasts. J Nucl Med. 2018 [cited 2024 Jun 11];59:1423–9. 10.2967/JNUMED.118.210435. 10.2967/jnumed.118.210435 PMC6126438 29626120

[9] Huizing DMV, Koopman D, van Dalen JA, Gotthardt M, Boellaard R, Sera T, et al. Multicentre quantitative 68Ga PET/CT performance harmonisation. EJNMMI Phys. 2019 [cited 2024 Jun 17];6:1–9. 10.1186/S40658-019-0253-Z/FIGURES/4. 10.1186/s40658-019-0253-z PMC6841905 31705215

[10] Wasserthal J, Breit HC, Meyer MT, Pradella M, Hinck D, Sauter AW, et al. TotalSegmentator: robust segmentation of 104 anatomic structures in CT images. Radiol Artif Intell. 2023 [cited 2024 Feb 14];5. 10.1148/RYAI.230024/ASSET/IMAGES/LARGE/RYAI.230024.VA.JPEG. 10.1148/ryai.230024 PMC10546353 37795137

[11] Janmahasatian S, Duffull SB, Ash S, Ward LC, Byrne NM, Green B. Quantification of lean bodyweight. Clin Pharmacokinet. 2005 [cited 2024 May 29];44:1051–65. 10.2165/00003088-200544100-00004/FIGURES/6. 10.2165/00003088-200544100-00004 16176118

[12] Pan F, Ye T, Sun P, Gui S, Liang B, Li L, et al. Time course of lung changes at chest CT during recovery from Coronavirus disease 2019 (COVID-19). Radiology. 2020 [cited 2024 Jun 11];295:715–21. 10.1148/RADIOL.2020200370/ASSET/IMAGES/LARGE/RADIOL.2020200370.FIG5.JPEG. 10.1148/radiol.2020200370 PMC7233367 32053470

[13] Telo S, Farolfi A, Castellucci P, Antonacci F, Solli P, Mosconi C, et al. A case of [68Ga]Ga-FAPI-46-avid and [18F]F-FDG-negative COVID-19 pneumonia sequelae. Eur J Nucl Med Mol Imaging. 2022 [cited 2024 Jun 10];49:2452–3. 10.1007/S00259-022-05720-0/METRICS. 10.1007/s00259-022-05720-0 PMC8854481 35179626

[14] Sviridenko A, Boehm A, Di Santo G, Uprimny C, Nilica B, Fritz J, et al. Enhancing clinical diagnosis for patients with persistent pulmonary abnormalities after COVID-19 infection: the potential benefit of 68Ga-FAPI PET/CT. Clin Nucl Med. 2022 [cited 2024 Jun 10];47:1026–9. 10.1097/RLU.0000000000004437. 10.1097/RLU.0000000000004437 PMC9653058 36257062

[15] Kastrati K, Nakuz TS, Kulterer OC, Geßl I, Simader E, Mrak D, et al. FAPi PET/CT for assessment and visualisation of active myositis-related interstitial lung disease: a prospective observational pilot study. 2024 [cited 2025 Mar 31]. 10.1016/j.eclinm.2024.102598. 10.1016/j.eclinm.2024.102598 PMC11019096 38633577

[16] Bergmann C, Distler JHW, Treutlein C, Tascilar K, Müller AT, Atzinger A, et al. 68Ga-FAPI-04 PET-CT for molecular assessment of fibroblast activation and risk evaluation in systemic sclerosis-associated interstitial lung disease: a single-centre, pilot study. Lancet Rheumatol. 2021;3:e185–94. 38279381 10.1016/S2665-9913(20)30421-5

[17] Bahtouee M, Jafari E, Khazaei M, Aram N, Amini A, Jokar N, et al. Exploring the potential value of [68Ga]Ga-FAPI-46 PET/CT for molecular assessment of fibroblast activation in interstitial lung disease a single-center pilot study. Clin Nucl Med. 2024 [cited 2025 Jan 22]. 10.1097/RLU.0000000000005505. 10.1097/RLU.0000000000005505 39466620

[18] Appelman B, Charlton BT, Goulding RP, Kerkhoff TJ, Breedveld EA, Noort W, et al. Muscle abnormalities worsen after post-exertional malaise in long COVID. Nat Commun. 2024;15:1–15. 38177128 10.1038/s41467-023-44432-3 PMC10766651

[19] Kessler L, Ferdinandus J, Hirmas N, Zarrad F, Nader M, Kersting D, et al. Pitfalls and common findings in 68Ga-FAPI PET: a pictorial analysis. J Nucl Med. 2022 [cited 2025 Jan 22];63:890–6. 10.2967/JNUMED.121.262808. 10.2967/jnumed.121.262808 PMC9157730 34620730

[20] Hope TA, Calais J, Goenka AH, Haberkorn U, Konijnenberg M, McConathy J, et al. SNMMI procedure standard/EANM practice guideline for fibroblast activation protein (FAP) PET. J Nucl Med. 2025 [cited 2025 Jan 22];66:26–33. 10.2967/JNUMED.124.269002. 10.2967/jnumed.124.269002 PMC11705787 39572227

[21] Sansores RH, Ramírez-Venegas A, Montiel-Lopez F, Domínguez-Arellano S, Alva-Lopez LF, Falfán-Valencia R, et al. Prolonged-release pirfenidone in patients with pulmonary fibrosis as a phenotype of post-acute sequelae of COVID-19 pneumonia. Saf Efficacy Respir Med. 2023;217:107362. 10.1016/j.rmed.2023.107362 37451648

[22] Duong-Quy S, Vo-Pham-Minh T, Tran-Xuan Q, Huynh-Anh T, Vo-Van T, Vu-Tran-Thien Q, et al. Post-COVID-19 pulmonary fibrosis: facts—Challenges and futures: a narrative review. Pulm Ther. 2023 [cited 2024 Jun 13];9:295–307. 10.1007/S41030-023-00226-Y/FIGURES/3. 10.1007/s41030-023-00226-y PMC10199290 37209374

[23] Glatting FM, Hoppner J, Liew DP, van Genabith A, Spektor AM, Steinbach L, et al. Repetitive Early 68Ga-FAPI PET acquisition comparing 68Ga-FAPI-02, 68Ga-FAPI-46, and 68Ga-FAPI-74: Methodologic and diagnostic implications for malignant, inflammatory/reactive, and degenerative lesions. J Nucl Med. 2022 [cited 2025 Apr 8];63:1844–51. 10.2967/JNUMED.122.264069. 10.2967/jnumed.122.264069 PMC9730916 35618480

[24] Naeimi M, Choyke PL, Dendl K, Mori Y, Staudinger F, Watabe T, et al. Three-time-point PET analysis of 68Ga-FAPI-46 in a variety of cancers. J Nucl Med. 2023 [cited 2025 Apr 8];64:618–22. 10.2967/JNUMED.122.264941. 10.2967/jnumed.122.264941 PMC11927082 36357183

